# Supplementary figures and images for: Paramutation-like features of multiple natural epialleles in tomato
Source: BMC Genomics. 2018 Mar 20;19:203. doi: 10.1186/s12864-018-4590-4 (PMC5859443; doi:10.1186/s12864-018-4590-4)

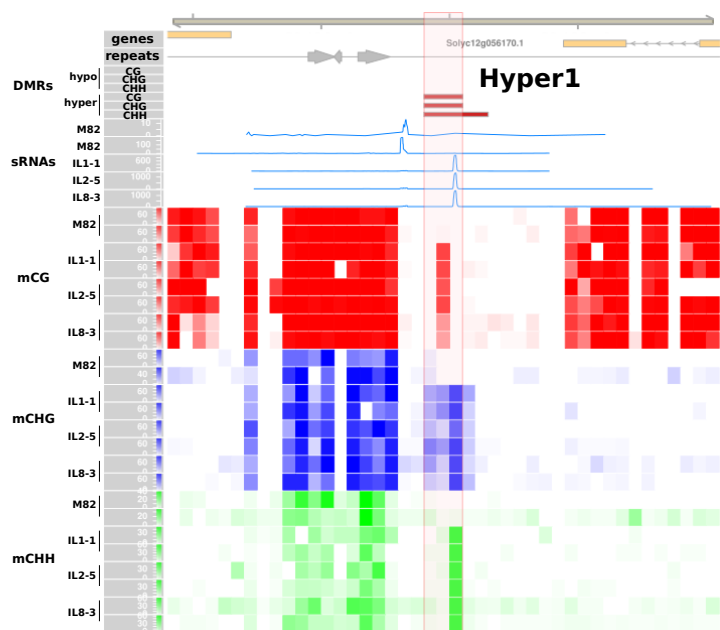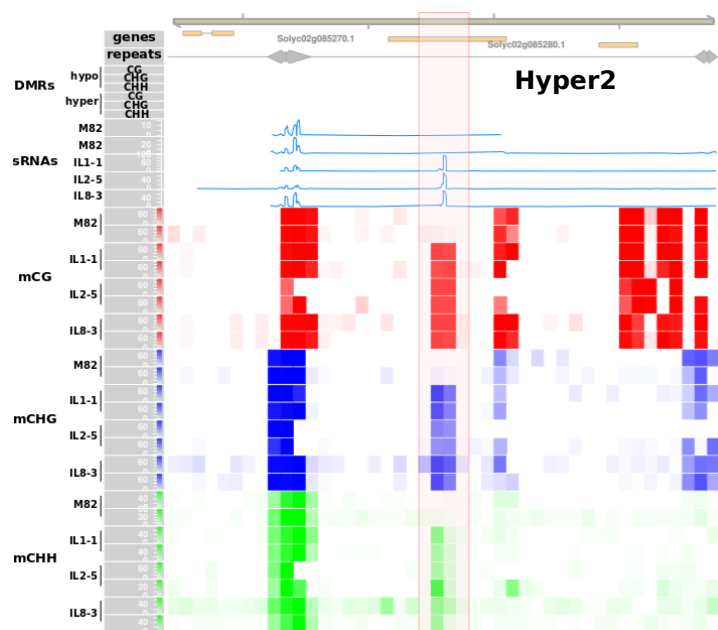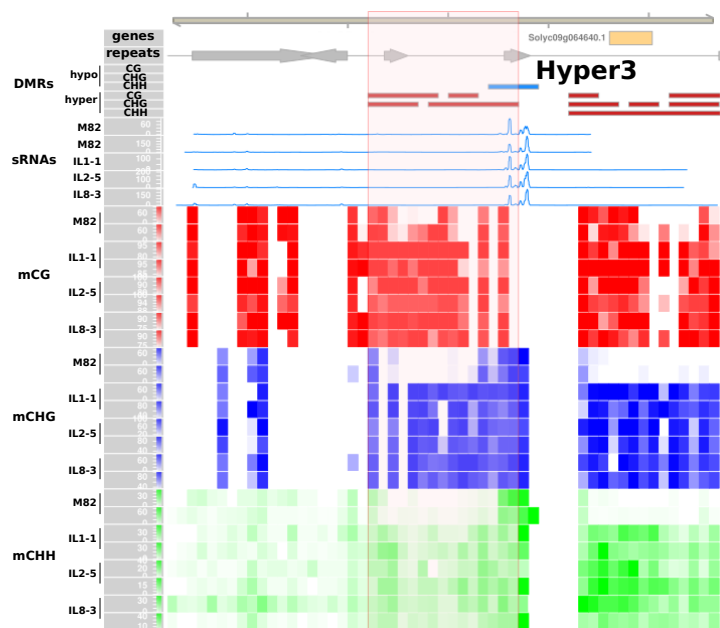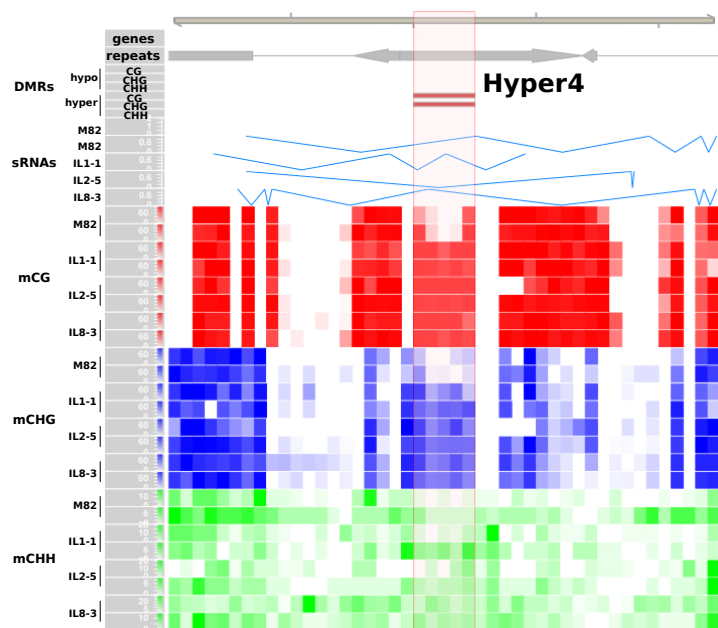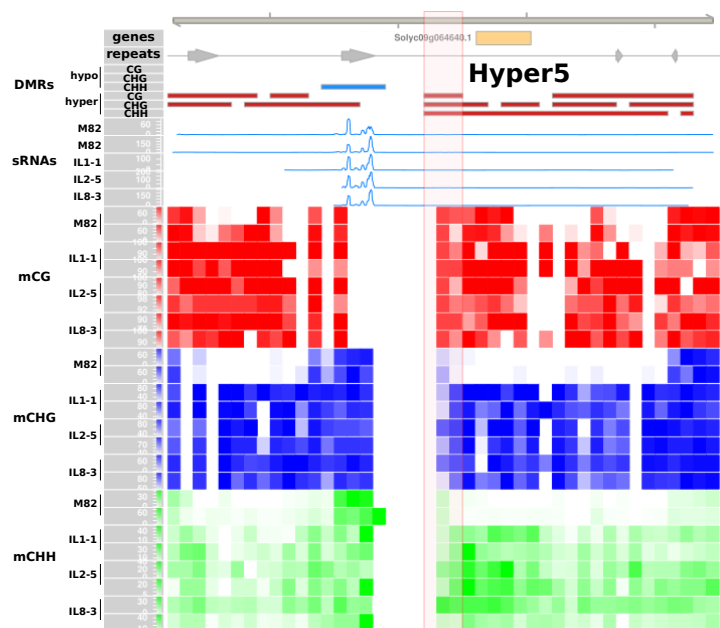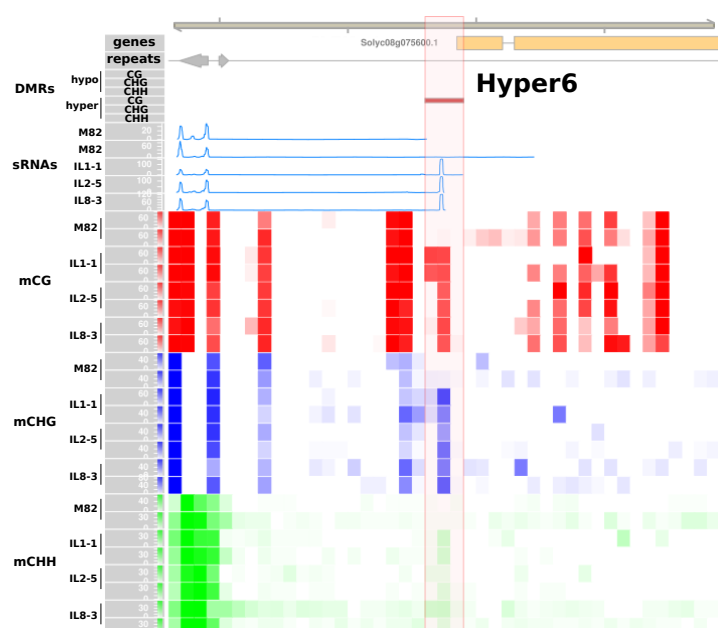

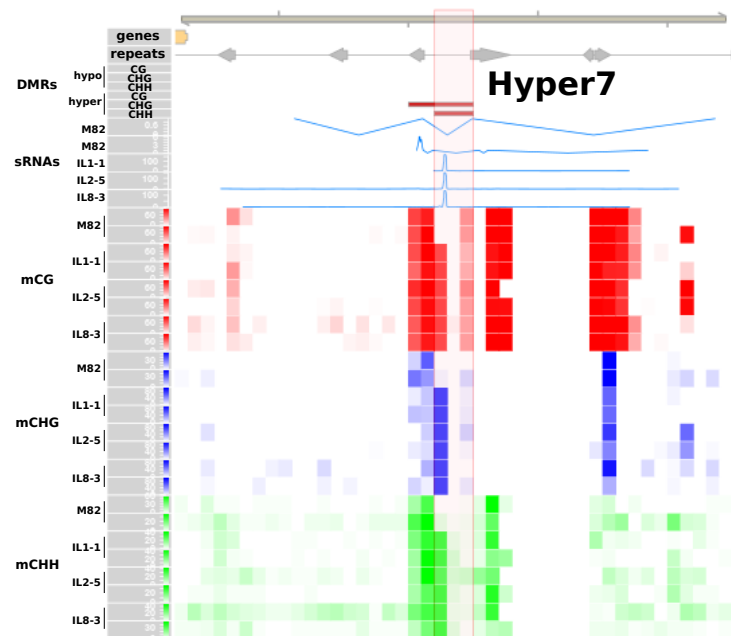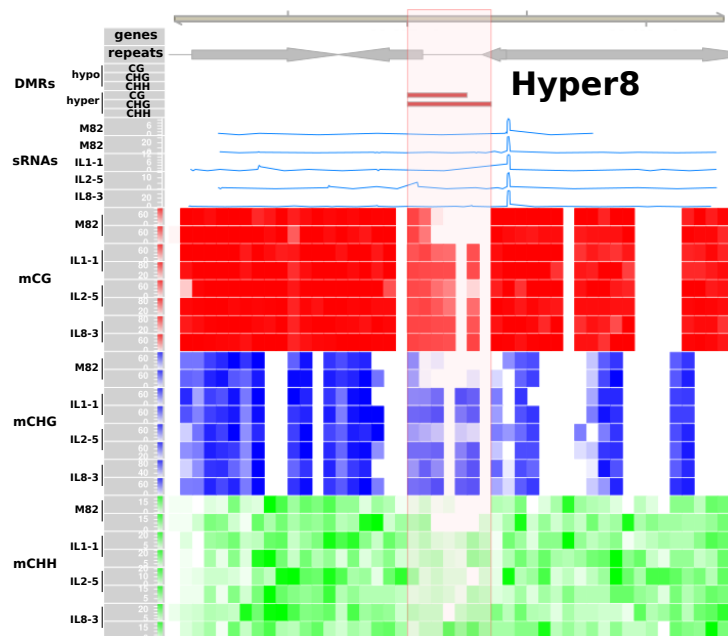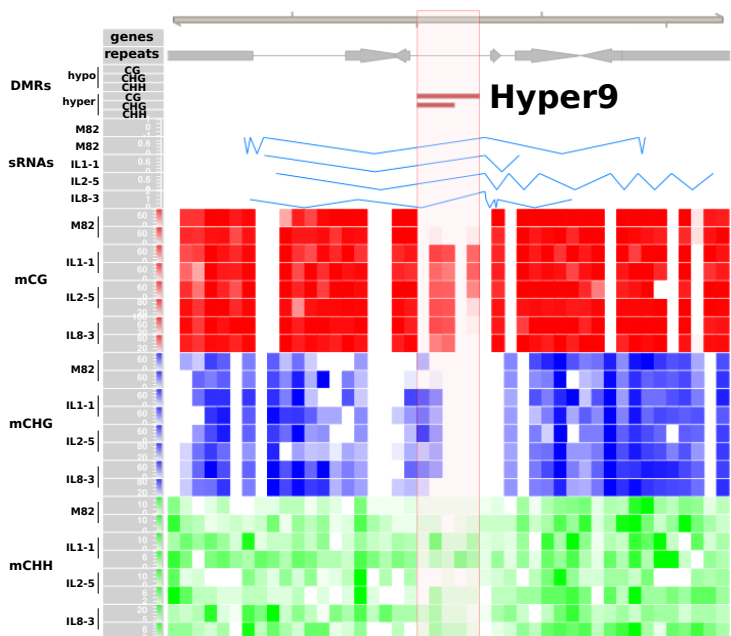

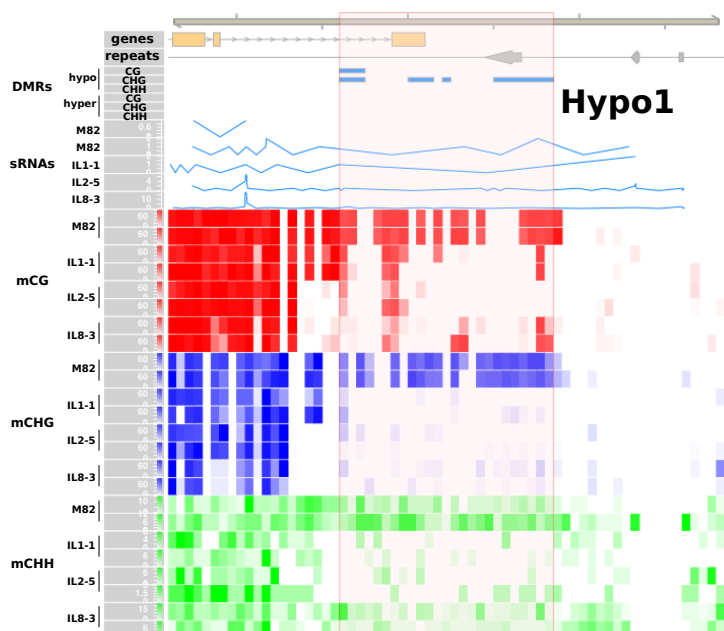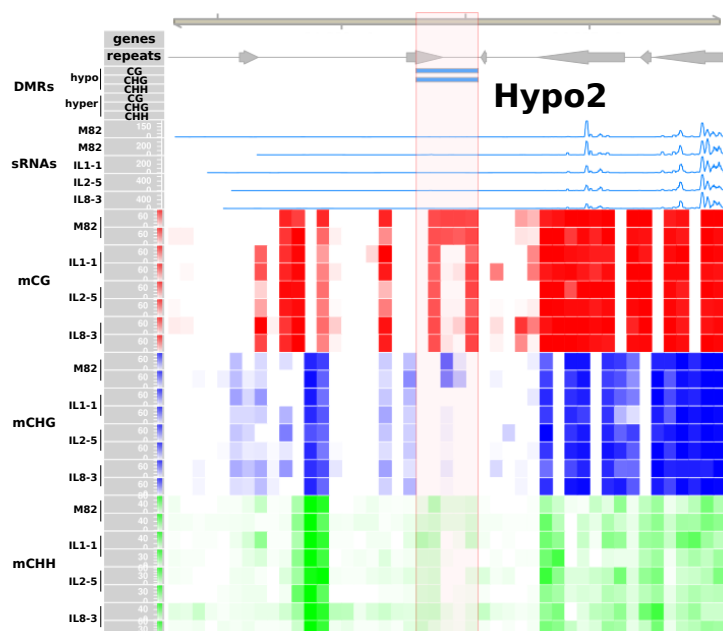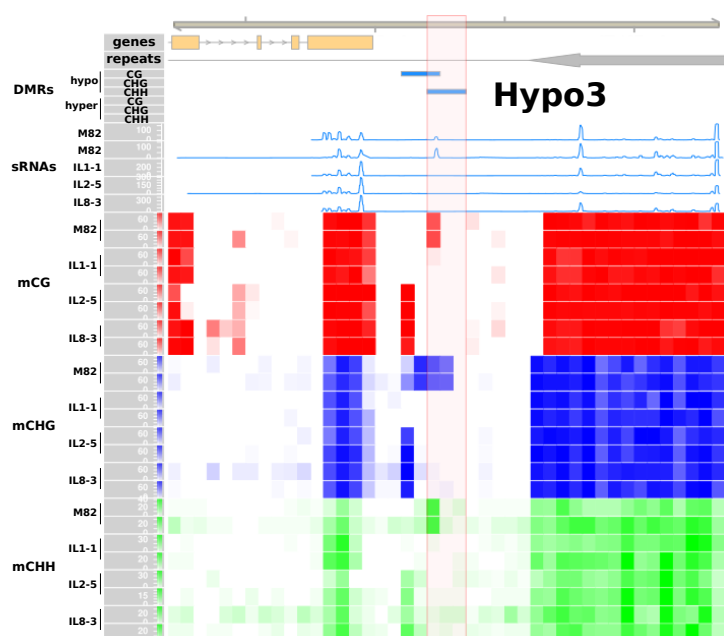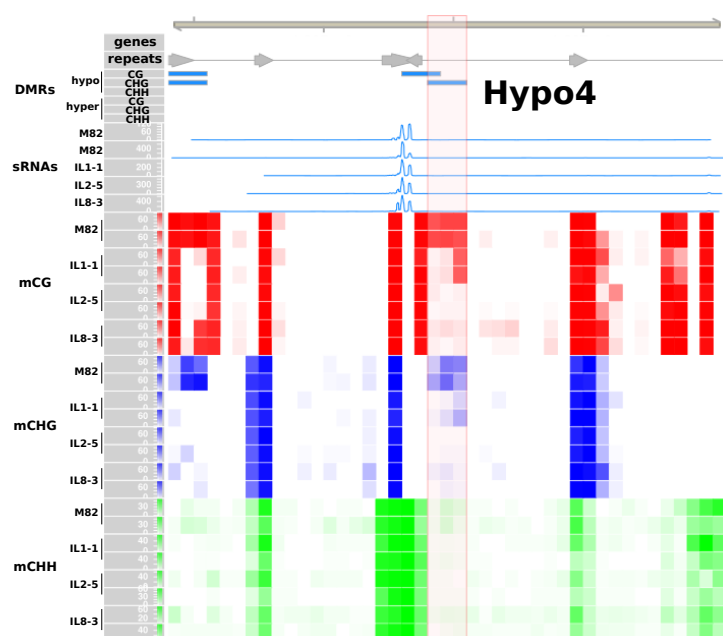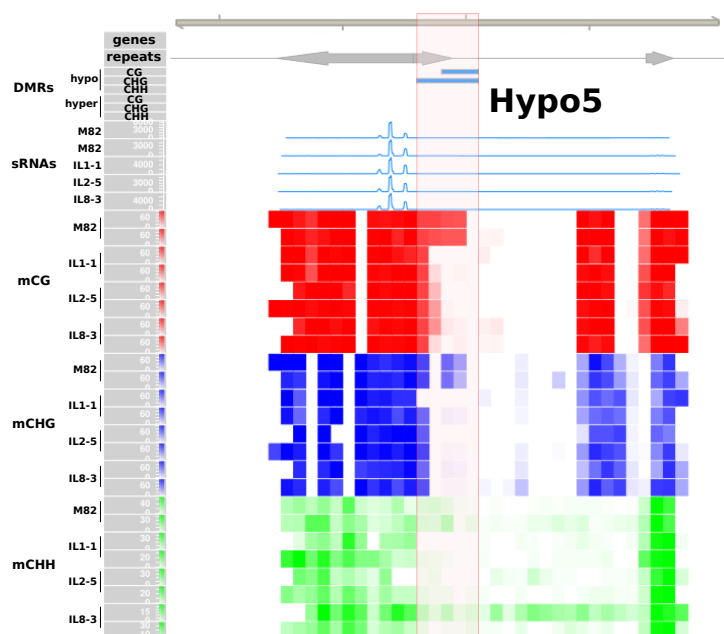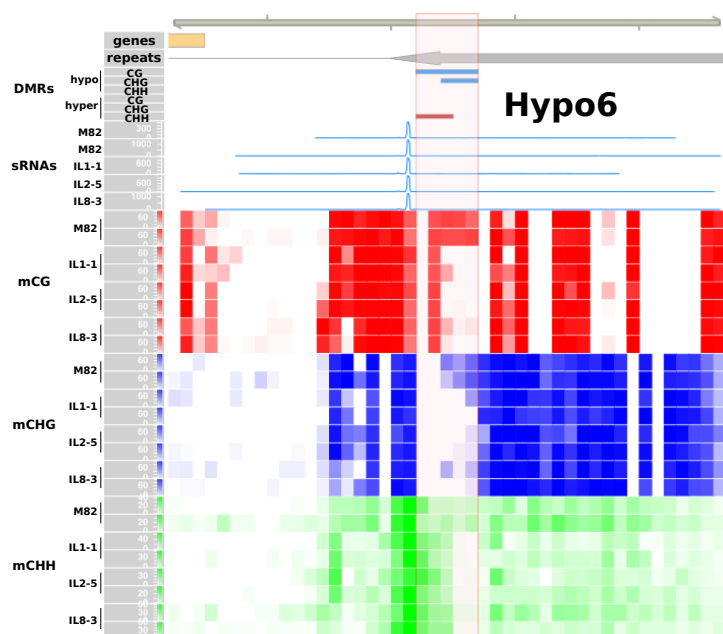

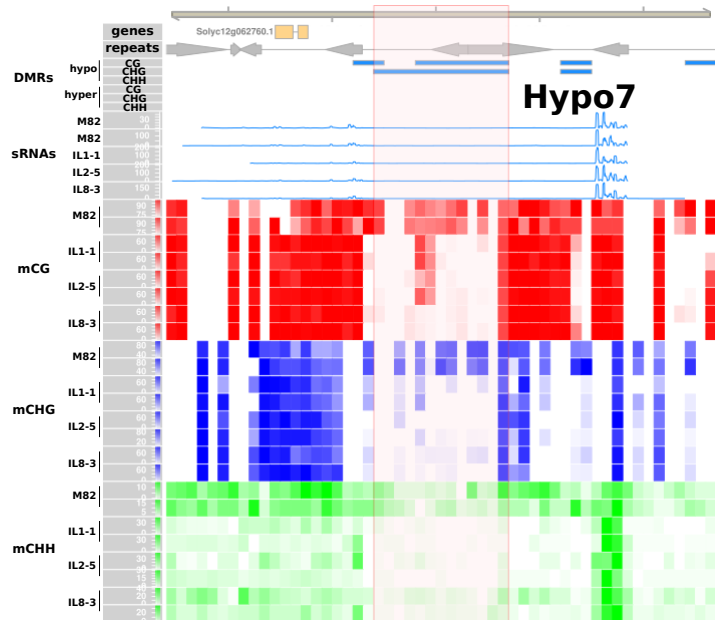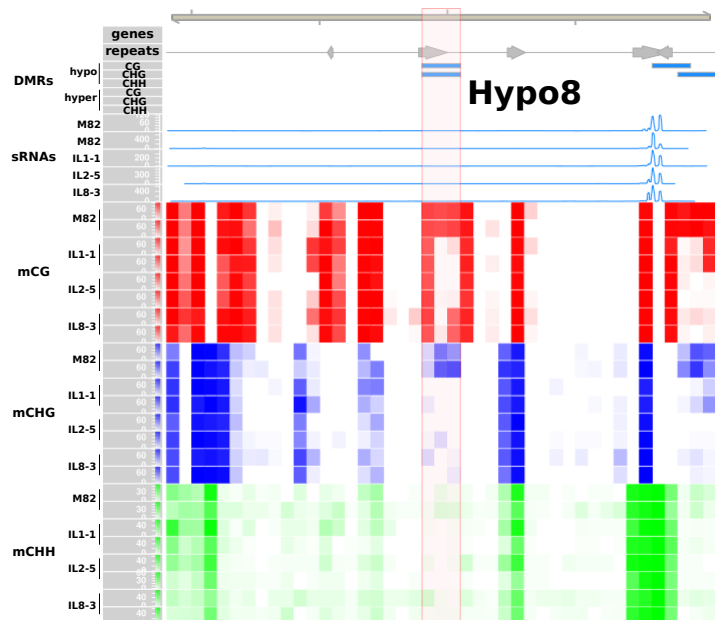

Supplement: Supplementary file 5 — Figure S1. Genomic position, small RNAs and DNA methylation of the IL DMRs Hyper1–9 and Hypo1–8. The selected DMR is highlighted in red, the plotted region includes 2 kb upstream and downstream. Genes: ITAG2.4 gene models. Repeats: RepeatMasker annotation. Hypomethylated regions shared between the three introgression lines are annotated in blue, and hypermethylated regions in red. sRNAs: coverage of sRNAs in seedlings. Methylation: percentage of methylated cytosines in 100-bp regions in each context (two replicates per genotype). (PDF 853 kb) [file 12864_2018_4590_MOESM5_ESM.pdf]

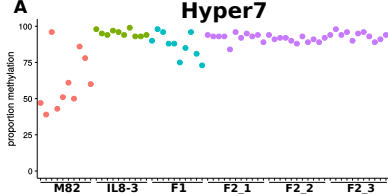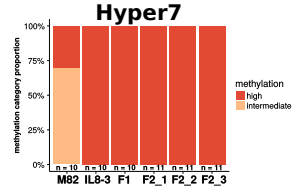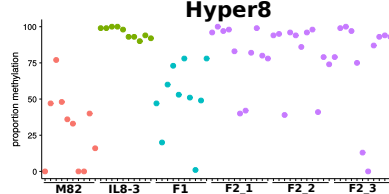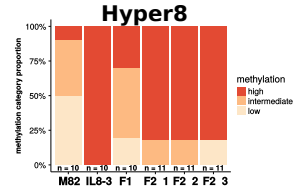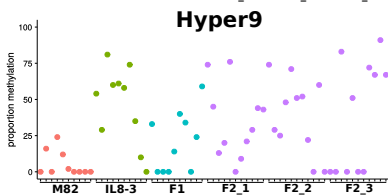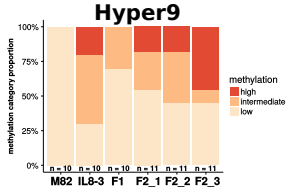

● M82  
● IL8-3  
● F1  
● F2

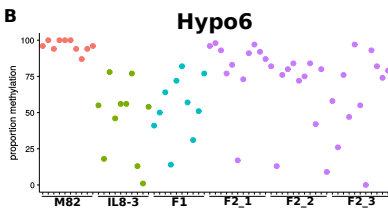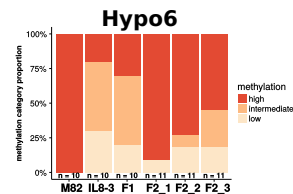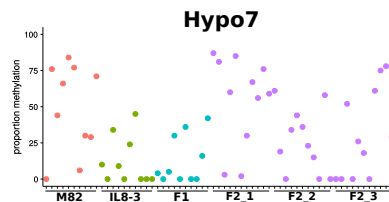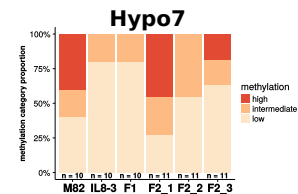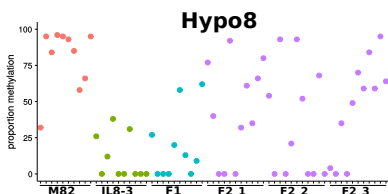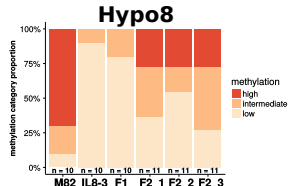

Supplement: Supplementary file 7 — Figure S3. Segregation of DNA methylation patterns by McrBC. These regions show variable methylation in the parental controls M82 and IL8–3. (A) Hypermethylated DMRs Hyper7–9. (B) Hypomethylated DMRs Hypo6–8. For each region, the left hand panel shows the results of methylation analysis by McrBC for individual plants. This information is collated in the right hand panel with the splitting of the F2 s according to their F1 parent. Low methylation: ≤ 33%. Intermediate: > 33% and ≤ 66%. High: > 66%. (PDF 135 kb) [file 12864_2018_4590_MOESM7_ESM.pdf]

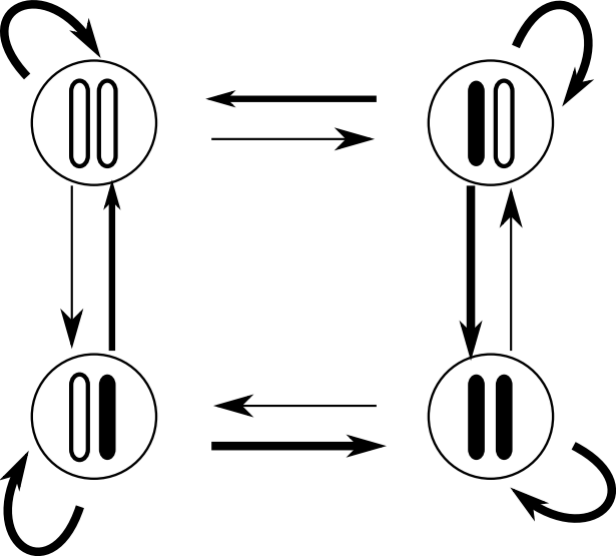

Supplement: Supplementary file 8 — Figure S4. Schematic of possible epiallelic transitions for one locus in a diploid state with unmethylated (white) and methylated (black) epialleles. There are four possible epigenetic configurations. Recurrent (curved) arrows represent the propensity to conserve the current state, from the action of local positive feedback loops maintaining methylation/unmethylation. Transition (straight) arrows between states represent spontaneous epimutations and, when starting from epigenetically heterozygous states, paramutation-like interactions. The epigenetic state of a genome may be seen as the result of such a stochastic process at each cell/organism generation. (PDF 2 kb) [file 12864_2018_4590_MOESM8_ESM.pdf]

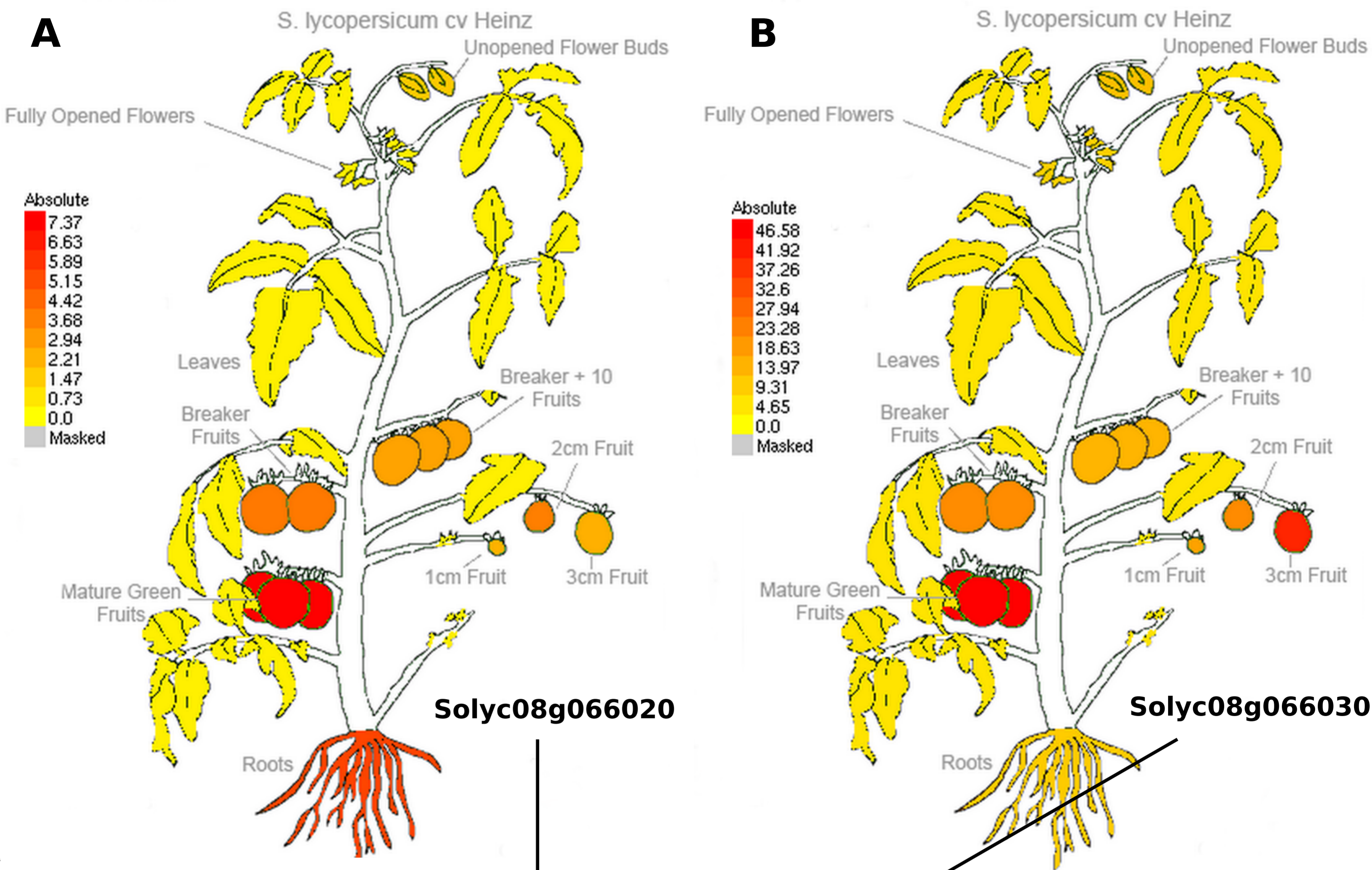

**C**

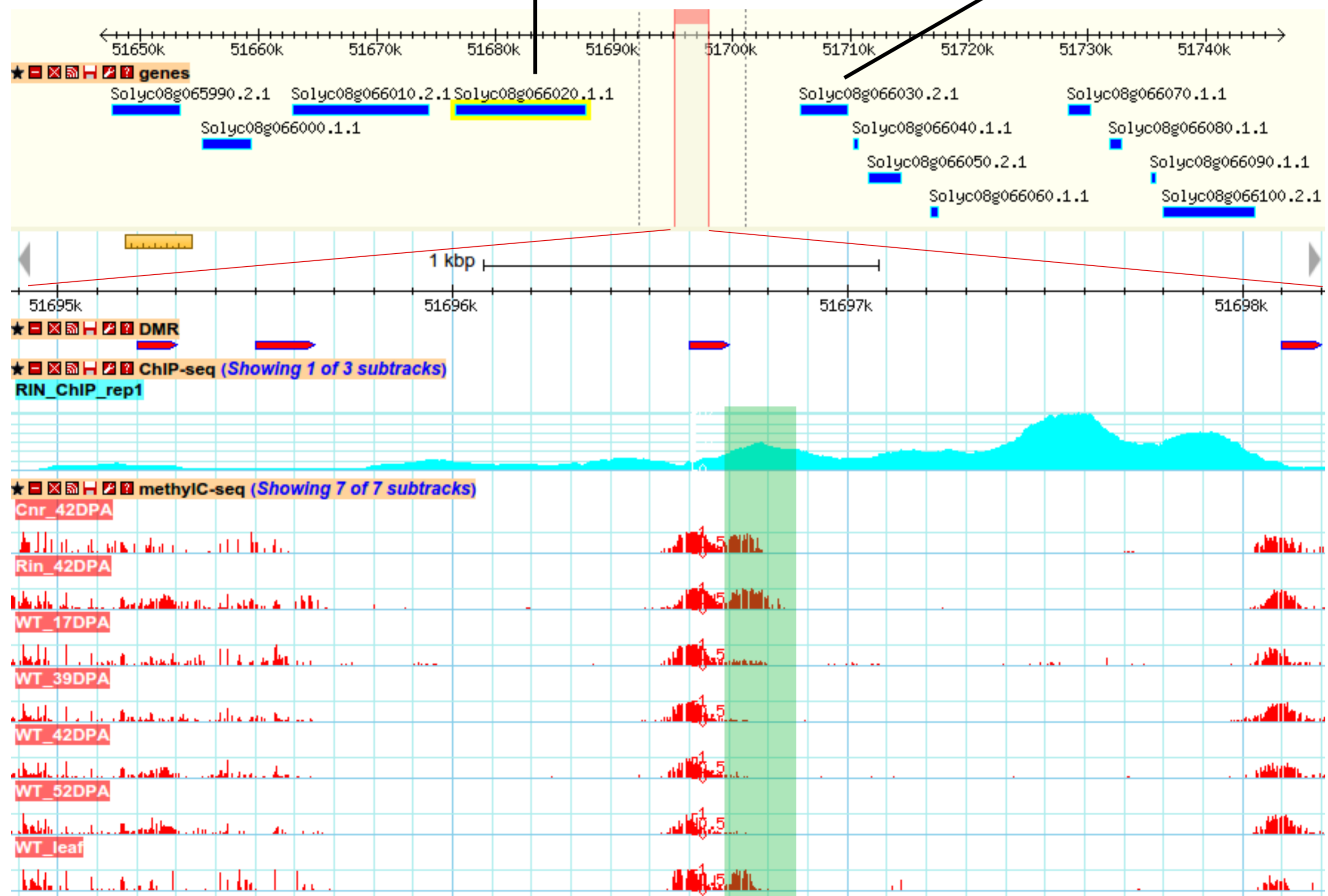

**RIN binding and demethylation**

**H06**

Supplement: Supplementary file 9 — Figure S5. Potential role of H06 in fruit ripening. Expression profiles across tissues of the two genes immediately downstream of H06, Solyc08g066020, encoding a Serine C-palmitoyltransferase like protein (A) and Solyc08g066030, encoding an unknown protein (B). Their expression peaks in mature green fruit. Data from [31] visualised on the eFP Browser [41] at http://bar.utoronto.ca/efp_tomato/. Copyright permissions for the re-use of the tomato drawings kindly granted by Prof. Provart. (C) Demethylation of the 3′ end of H06 during fruit ripening correlates with the binding of the RIN transcription factor at this locus. RIN binding, demethylation and ripening are compromised in the rin (ripening inhibitor) and cnr (colorless non-ripening) mutants. Data from [30]. (PDF 971 kb) [file 12864_2018_4590_MOESM9_ESM.pdf]
