# Supplementary material for: Paramutation-like features of multiple natural epialleles in tomato
Source: BMC Genomics. 2018 Mar 20;19:203. doi: 10.1186/s12864-018-4590-4 (PMC5859443; doi:10.1186/s12864-018-4590-4)
Supplement: Supplementary file 6 — Figure S2. A) Distribution of zero-count data for the genes differentially expressed between M82 and ILs (70 samples: 40 M82 and 30 ILs, data from [17]). B) Differential expression of genes whose promoters overlap shared IL DMRs selected for segregation analysis. Hypermethylation of Solyc09g064640’s promoter (Hyper3 and Hyper5) is associated with decreased expression in introgression lines. Solyc09g064640 codes for a protein related to the family with sequence similarity 65 member A (FAM65A). Solyc04g025030, encoding a MADS-box transcription factor similar to Agamous-like MADS-box protein AGL62, is associated with Hyper4, and Solyc06g075840, encoding a putative zinc-binding protein of the Yippee family, with Hypo3. Normalised counts in seedlings (40 M82 and 30 ILs, data from [17]). (PDF 64 kb) [file 12864_2018_4590_MOESM6_ESM.pdf]

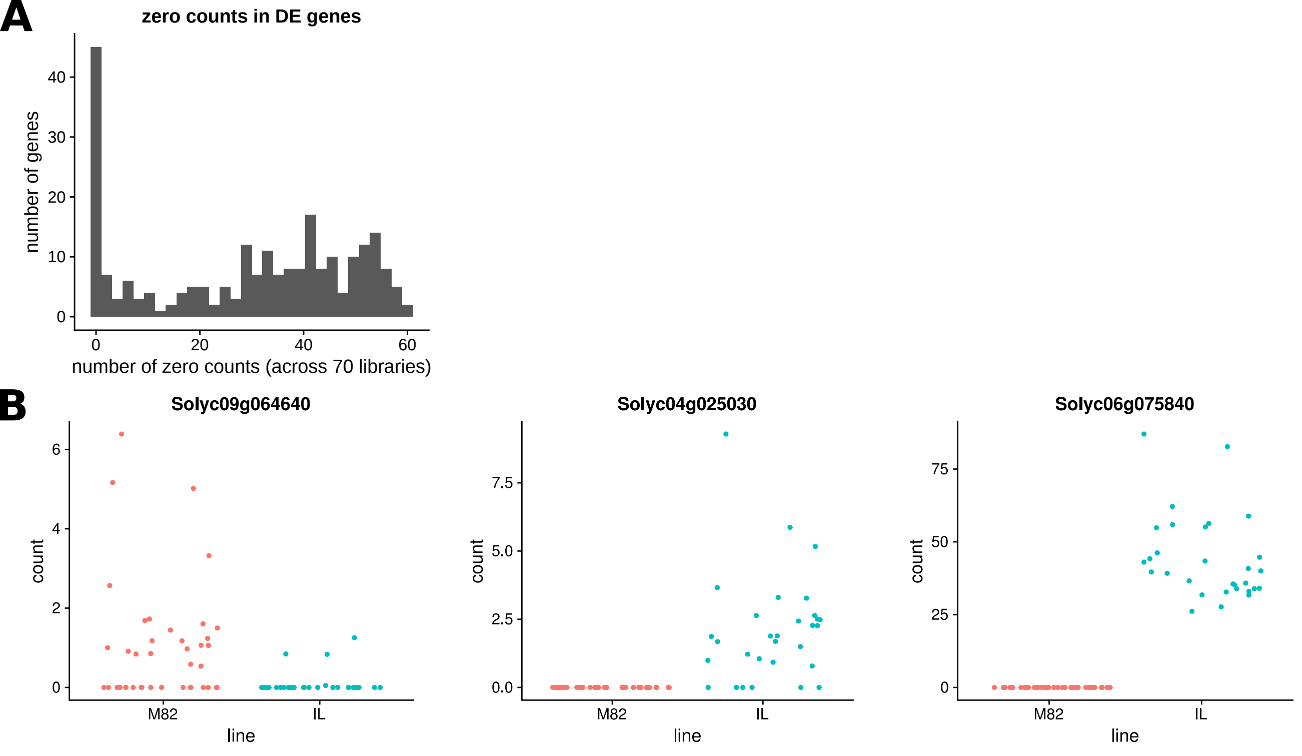
A
zero counts in DE genes
number of genes
number of zero counts (across 70 libraries)
0
10
20
30
40
60
B
Solyc09g064640
Solyc04g025030
Solyc06g075840
count
line
M82
IL
0
2
4
6
0.0
2.5
5.0
7.5
25
50
75
